# Exploring the impact of childhood maltreatment and BPD on impulsivity in crimes of passion

**DOI:** 10.3389/fpsyt.2023.1159678

**Published:** 2023-07-17

**Authors:** Michelle Jin, Zhongrui Wang, Ying Zhou, Jie Zhong

**Affiliations:** ^1^Beijing Key Laboratory of Behavior and Mental Health, Clinical and Health Psychology Department, School of Psychological and Cognitive Science, Peking University, Beijing, China; ^2^Center for Psychological Health Education, Xihua University, Chengdu, China; ^3^Shenzhen Prison, Shenzhen, China

**Keywords:** impulsivity, crimes of passion, borderline personality disorder, childhood maltreatment, inmates

## Abstract

**Background:**

Crimes of passion, characterized as unpremeditated impulsive aggression, have garnered increasing attention in recent years. Impulsivity, a major factor in crimes of passion, is also a common feature of various health conditions, including Borderline Personality Disorder (BPD). Childhood maltreatment is considered a significant precursor to BPD and is closely related to impulsivity. Although prior research has affirmed the relationship between impulsivity, childhood maltreatment, BPD, and criminal behavior, few studies have examined these variables’ interconnections within the context of crimes of passion. This study seeks to explore the relationship between childhood maltreatment, BPD, and impulsivity in crimes of passion, assessing the former’s impact on the latter.

**Method:**

Our research examined 133 crimes of passion offenders and 149 other offenders from the Shenzhen male prison in China. All 282 participants completed The Abbreviated Version of The Barratt Impulsiveness Scale (ABIS), The UPPS Impulsivity Scale (Urgency, Premeditation, Perseverance, Sensation Seeking), The McLean Screening Instrument for Borderline Personality Disorder (MSI-BPD), and The Childhood Trauma Questionnaire (CTQ).

**Results:**

Our findings indicated that (1) crimes of passion offenders scored significantly higher in emotional neglect, physical neglect, and overall childhood maltreatment than did other offenders, and childhood maltreatment scores were notably higher in the high BPD trait group. (2) Crimes of passion offenders demonstrated significantly elevated impulsivity in attention and nonplanning scales compared to other offenders. Impulsivity scores across all scales were also significantly higher in the high BPD trait group. (3) Emotional neglect was found to significantly influence the urgency scale in crimes of passion offenders. An interaction effect was noted between physical abuse and high BPD traits, leading to increased impulsivity in crimes of passion offenders.

**Conclusion:**

This study underscores the predictive roles of childhood maltreatment and BPD in determining impulsivity within the context of crimes of passion.

## Introduction

Crimes of passion, also known as impulsive crimes, are characterized by sudden, emotionally-charged acts of transgression, typically absent premeditation (1). Interpretations of this term vary among researchers, some focusing on crimes committed under the influence of intense emotions, and others emphasizing crimes without premeditation. In the present study, we adopt the latter perspective, shedding light on crimes propelled by potent emotions without forethought.

The discourse around crimes of passion spans law, criminology, and psychology (2, 3). Past investigations have connected repeated impulsive aggression with a stable personality tendency, defined within the domain of intermittent explosive disorder (IED) (4, 5). However, this definition, bearing conspicuous pathological traits, should be limited to individuals with psychological disorders.

More recent research has revised the concept of crimes of passion, devising a checklist delineating seven crucial factors: inducement, object, violence, passion, loss of control, time, and exclusion. This checklist lays the groundwork for a deeper understanding of the psychological mechanisms underlying crimes of passion. Our research utilizes this tool for screening offenders involved in crimes of passion (6).

At the core of crimes of passion lies impulsive, aggressive behavior, closely associated with the psychological concept of impulsivity (7). Impulsivity refers to a tendency to respond rapidly to internal or external stimuli, with individuals reacting so swiftly and without forethought that they often overlook potential adverse consequences (7). Consequently, uncovering factors contributing to offenders’ impulsivity is critical for comprehending crimes of passion.

Childhood maltreatment, encompassing physical, emotional, and sexual abuse, as well as emotional and physical neglect, constitutes a significant risk factor for various types of crimes (8). As a traumatic experience, maltreatment in childhood can influence an individual’s emotional and stress responses, pushing them towards maladaptive emotional regulation strategies, thereby escalating impulsivity and criminal propensity (9, 10).

Childhood maltreatment can significantly hinder a child’s development and elevate the risk of adult mental disorders, notably Borderline Personality Disorder (BPD) (11). Despite existing research on the links between childhood maltreatment, BPD, and criminal behavior (12), few studies have delved into their pathological influence on crimes of passion. The present study aims to bridge this gap by elucidating the relationships between childhood maltreatment, BPD, impulsivity, and crimes of passion.

BPD is recognized as a chronic psychiatric disorder typified by impulsivity, mood instability, unstable interpersonal relationships, and suicidal behaviors. It is accompanied by emotional, impulsive, interpersonal, and cognitive symptoms (13). Prior studies have elucidated the relationship between BPD, impulsivity, and violent crime (14, 15), indicating that individuals with prominent BPD traits often exhibit heightened emotional reactivity to external stimuli and struggle more with impulsivity control, thereby leading to heightened aggressive behavior. Consequently, we posit that a pronounced BPD trait could be a significant risk factor for impulsivity in perpetrators of crimes of passion.

Earlier studies have suggested that the impulsivity demonstrated by those who commit crimes of passion is predominantly manifested as urgency and lack of perseverance (16). The present study aims to corroborate and augment these findings while further investigating the underlying factors contributing to impulsivity in offenders of crimes of passion. Simultaneously, we examine the prevalence of childhood maltreatment and high BPD traits among these offenders and their respective impacts on impulsivity within the context of crimes of passion.

Informed by existing research, we propose three hypotheses: (1) Childhood maltreatment and high BPD traits are strongly associated with crimes of passion; (2) Offenders with a history of childhood maltreatment and high BPD traits exhibit greater impulsivity within the crimes of passion cohort; and (3) Childhood maltreatment and high BPD traits interact to heighten impulsivity levels in perpetrators of crimes of passion.

## Methods

### Participants

Utilizing the crimes of passion checklist as developed by Huang (6), we engaged 500 inmates from a male prison in mainland China. This sample included 250 offenders implicated in crimes of passion and 250 perpetrators of other offenses. Four correctional officers participated in the evaluation of files and questionnaires. The checklist exhibited good internal consistency reliability among the inmates, ranging from 0.90 to 0.98, and rater reliability was noted at 0.81, demonstrating good reliability and validity.

Inclusion was based on voluntary participation. From the initial group, 358 participants completed the questionnaires. Following the exclusion of surveys with missing values and inconsistent answers, a total of 282 valid questionnaires were retained. These comprised 133 from offenders involved in crimes of passion and 149 from those convicted of other offenses. All participants were males, aged between 20 and 63 years (M = 36.4, SD = 9.04).

#### Ethical approvals and consent

The present study secured approval from the Academic Committee of the School of Psychological and Cognitive Sciences at Peking University. It also obtained an ethics review certificate from the Shenzhen Prison in Guangdong Province, mainland China. All participants provided their written informed consent prior to questionnaire completion.

#### Incentives

In accordance with prison guidelines prohibiting monetary rewards, participants received a prize of their choice upon questionnaire completion. These incentives included notebooks, pencils, postcards, T-shirts, snacks, and cigarettes.

### Measures

The survey instrument consisted of five components: basic demographic information, the Crimes of Passion Checklist, the Childhood Trauma Questionnaire (CTQ), the Mclean Screening Instrument for Borderline Personality Disorder (MSI-BPD), the Abbreviated Version of the Barratt Impulsiveness Scale (ABIS), and the UPPS Impulsive Behavior Scale (UPPS).

#### Crimes of passion checklist (peer assessment version)

The Crimes of Passion Checklist, developed and revised by Huang (6), assesses the nature of an inmate’s offense based on case files and records. Comprising nine components, this checklist incorporates three subjective criteria, three objective criteria, and three exclusion criteria. Evaluated using a forced-choice method, it demonstrates strong credibility and validity. Professionally trained correctional officers administered the checklist in this study.

#### The Mclean screening instrument for borderline personality disorder (MSI-BPD)

The MSI-BPD, developed by Bernstein et al. (17), assesses participants’ BPD traits. The scale, derived from the diagnostic criteria for BPD in the Diagnostic and Statistical Manual of Mental Disorders, Fourth Edition (DSM-IV), contains 10 items. In this study, we used the scale not for BPD diagnosis but to investigate potential relationships between BPD symptoms and other variables. We followed other studies (Crow and Levy, 2019) (18) and used five points as the cutoff value for distinguishing high and low BPD symptom groups. The scale demonstrates good sensitivity (0.81) and specificity (0.85), particularly among younger subjects (adolescents and young adults with a history of childhood maltreatment).

#### Childhood Trauma Questionnaire (CTQ)

Bernstein et al.’s CTQ (17) assesses childhood maltreatment. This retrospective self-reporting scale measures various forms of childhood trauma, including physical, emotional, and sexual abuse, emotional neglect, and physical neglect. Suitable for adolescents and adults, it contains 28 items, including three validity items. The CTQ boasts good reliability and validity, with test–retest reliability of 0.95 and internal consistency reliability of 0.88.

#### Impulsivity measurement

Considering the multidimensional nature of impulsivity, this study used two different scales to measure impulsivity: the UPPS Impulsive Behavior Scale (19) and the abbreviated version of the Barratt Impulsiveness Scale (ABIS) (20).

The UPPS Impulsive Behavior Scale consists of 45 questions with four dimensions of impulsivity: urgency, premeditation, perseverance, and sensation seeking. Previous studies have shown that the scale uses overlapping but different concepts for measurement and has good reliability and validity (19). ABIS consists of 13 questions, which is an abbreviated version of the original Barratt Impulsiveness Scale (BIS). The ABIS can be used to measure three independent but interrelated dimensions of impulsivity: attention, motor and nonplanning. The ABIS also has good reliability and validity. The internal consistency reliability of the three dimensions of the scale are: 0.67 for attention, 0.75 for motion, and 0.74 for nonplanning (21).

### Data analysis

IBM SPSS Statistics, version 22.0, was used for statistical analysis. Initially, quantitative variables were described with absolute and relative frequencies (percentages). Chi-square tests were subsequently applied for comparisons between the quantitative variables. Considering the continuous nature of impulsivity, independent sample t-tests were employed to compare impulsivity differences among inmate groups. Pearson correlations tested the relationships among each variable: impulsivity, childhood maltreatment, and BPD score. For categorical variables, such as crime type, Spearman correlation was used. Multivariate ANOVA assessed the interaction of BPD and childhood maltreatment on impulsivity. Lastly, hierarchical regression analysis determined the predictive role of impulsivity in crimes of passion offenders.

## Results

### Descriptive statistic

A total of 282 participants were divided into different groups based on their crime type and BPD traits for the analysis. According to MSI-BPD scores, 48 participants were classified into the high BPD symptom group and 234 participants into the low BPD symptom group. Statistical results are depicted in Table 1. Chi-square tests revealed that the crimes of passion group experienced significantly more childhood maltreatment compared to the other group (χ^2^ = 7.49, *df* = 1, *p* < 0.01), especially in emotional (χ^2^ = 8.84, *df* = 1, *p* < 0.01) and physical neglect (χ^2^ = 7.82 ~ 9.84, *df* = 1, *p* < 0.01). Moreover, the high BPD trait group was exposed to more childhood maltreatment than the low BPD trait group, except for sexual abuse (χ^2^ = 4.72–9.85, *df* = 1, *p* = 0.002–0.03).

**Table 1:** Ratio of childhood maltreatment among crime type and BPD traits.

Type of crime	BPD traits	Sample	Childhood maltreatment	Emotional	Physical	Sexual	Neglect E	Neglect P	None
	High	24	91.67%	4.17%	16.67%	20.83%	70.83%	70.83%	8.33%
			−22	−1	−4	−5	−17	−17	−2
Crimes of passion	Low	109	74.31%	1.83%	5.50%	10.09%	51.38%	59.63%	25.69%
			−81	−2	−6	−11	−56	−65	−28
	All	133	77.44%	2.26%	7.52%	12.03%	54.89%	61.65%	22.56%
			−103	−2	−10	−16	−73	−82	−30
	High	24	83.33%	8.33%	12.50%	16.67%	62.50%	70.83%	16.67%
			−20	−2	−3	−4	−15	−17	−4
Other	Low	125	58.40%	0.80%	1.60%	13.60%	33.60%	37.60%	41.60%
			−73	−1	−2	−17	−42	−47	−52
	All	149	62.42%	2.01%	3.36%	14.09%	38.26%	42.95%	37.48%
			−93	−3	−5	−21	−57	−64	−56

### *T*-test

Considering the multidimensional nature of impulsivity, the UPPS and ABIS scales were employed, with a total of 7 sub-scales: urgency, premeditation, perseverance, sensation seeking, attention, motor, nonplanning, and a UPPS total score. The independent sample *T*-test revealed significant differences between the crimes of passion group and the other group in the attention (*t* = 2.26, *p* < 0.05) and unplanning (*t* = 3.92, *p* < 0.001). No significant differences were found in the remaining impulsivity dimensions (*p* > 0.05). Results are shown in Table 2.

**Table 2:** Difference in impulsivity between crimes of passion offenders and other offenders.

Type of crime	Attention		Motor		Nonplanning		Premeditation		Urgency		Sensation seeking		Perseverance		UPPS total	
	Mean	SD	Mean	SD	Mean	SD	Mean	SD	Mean	SD	Mean	SD	Mean	SD	Mean	SD
Crimes of passion	2.33	0.61	1.81	0.62	2.60	0.77	3.29	2.87	4.01	3.70	3.42	3.45	3.02	2.28	13.73	8.31
Other	2.17	0.56	1.70	0.50	2.26	0.67	3.09	2.95	3.37	3.55	3.99	3.54	2.75	2.18	13.20	8.49

Participants were divided into high and low BPD trait groups based on their MSI-BPD scores. Due to the limited sample size, the present study chose to follow the method of Wang Yuyin et al. (22) dividing the high BPD trait group (subclinical group) based on a score of 5 and higher with a total of 48 participants; and the low BPD trait group with scores of 4 and lower, a total of 234 participants. We used an independent sample *t*-test to investigate the differences in impulsivity between the high BPD trait group and the low BPD trait group shown in Table 3. We found that the high BPD trait group showed higher impulsivity across all impulsivity scales than the low BPD trait group (*t* = 2.10 to 6.30, *p* = 0.001 to 0.036 < 0.05).

**Table 3:** Difference in impulsivity between BPD traits.

BPD traits	Attention		Motor		Nonplanning		Premeditation		Urgency		Sensation seeking		Perseverance		UPPS total	
	Mean	SD	Mean	SD	Mean	SD	Mean	SD	Mean	SD	Mean	SD	Mean	SD	Mean	SD
High	2.45	0.63	1.91	0.49	2.67	0.74	4.65	3.10	6.79	3.83	5.04	3.60	4.08	2.36	20.56	8.00
Low	2.21	0.57	1.72	0.57	2.37	0.73	2.89	2.78	3.03	3.24	3.45	3.43	2.62	2.12	12.00	7.72

### Correlation analysis

To further explore the associations among BPD traits, childhood maltreatment, and impulsivity in crimes of passion, we tested the correlations of various dimensions of impulsivity with the above variables (See Table 4). We found that crimes of passion showed a moderate correlation with nonplanning (*r* = 0.230, *p* < 0.001) and a weak correlation with attention (*r* = 0.134, *p* = 0.024 < 0.05). In addition, BPD traits had a strong correlation with urgency (*r* = 0.455, *p* < 0.001) and the UPPS total score (*r* = 0.452, *p* < 0.001); moderate correlations with total score of childhood maltreatment, premeditation, sensation seeking, and perseverance (*r* = 0.234 ~ 0.386, *p* < 0.001); and weak correlations with attention, motor, and nonplanning (*r* = 0.119 ~ 0.133, *p* = 0.025 ~ 0.046 < 0.05). Finally, we found that childhood maltreatment had a moderate correlation with UPPS impulsivity total score (*r* = 0.217, *p* < 0.001) and urgency (*r* = 0.221, *p* < 0.001), and a weak correlation with attentional impulsivity.

**Table 4:** Correlation between type of crime, BPD traits, childhood maltreatment and impulsivity.

	Attention	Motor	Nonplanning	Premeditation	Urgency	Sensation seeking	Perseverance	UPPS total
	*r*	*r*	*r*	*r*	*r*	*r*	*r*	*r*
Type of crime	0.134*	0.094	0.230***	0.034	0.088	0.082	0.061	0.032
BPD traits	0.133*	0.119*	0.132*	0.234***	0.456***	0.250***	0.262***	0.452***
Childhood maltreatment	0.121*	0.111	0.111	0.143*	0.221***	0.087	0.133*	0.217***

*p < 0.05, ***p < 0.001.

### Multivariate ANOVA

Multivariate ANOVA was used to further study the association of childhood maltreatment, BPD, and impulsivity in crimes of passion. We used the ABIS and UPPS impulsivity scales as dependent variables and divided the participants using BPD traits and childhood maltreatment types as variables.

**Figure 1:**
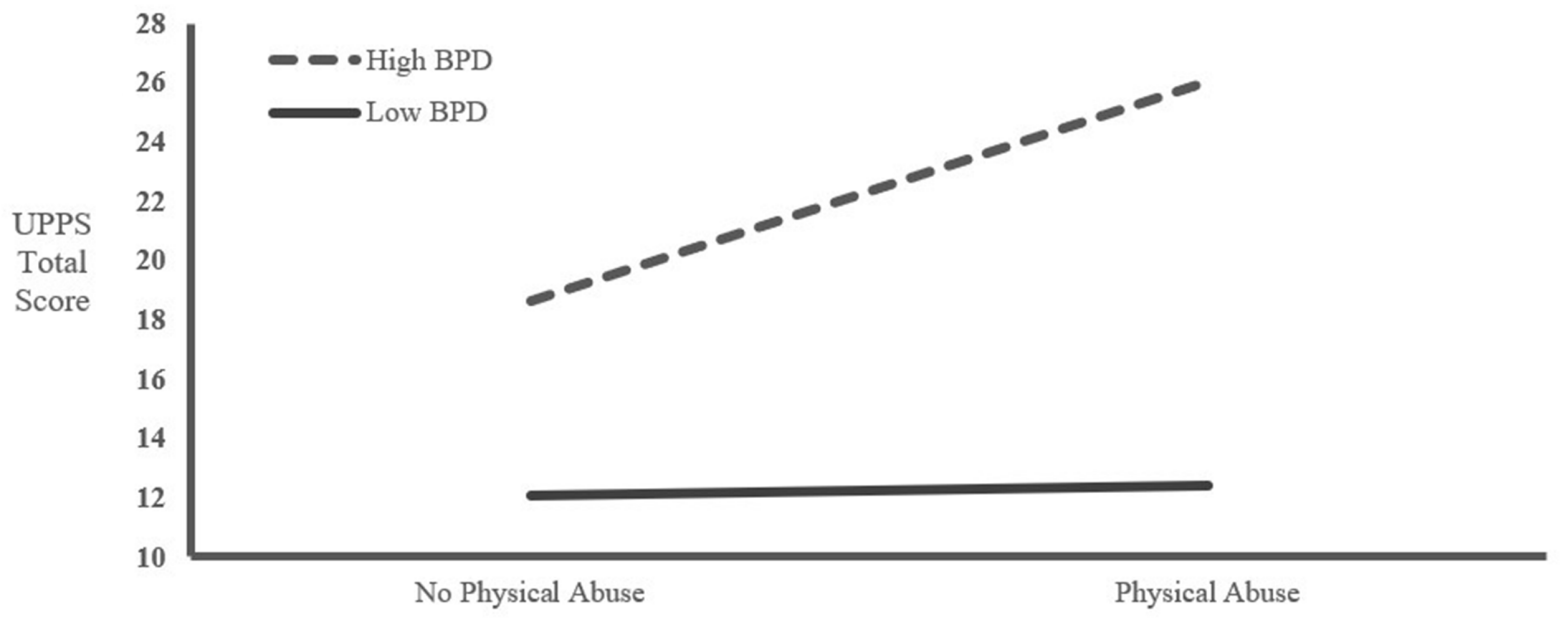
Interaction between high BPD trait and physical abuse on the UPPS total score.

The results of multivariate ANOVA are shown in Tables 5 6. We found that the high BPD trait group and physical abuse jointly affected the UPPS total score (*F* = 4.458, *p* = 0.037 < 0.05). In addition, the high BPD trait group and physical abuse significantly impacted the UPPS total score (*F* = 5.945, *p* = 0.025 < 0.05), while the low BPD trait group and physical abuse did not significantly impact impulsivity. This result suggested a significant interaction between high BPD traits and physical abuse affecting impulsivity (see Figure 1). Meanwhile, we found that emotional neglect showed a significant effect when predicting urgency (*F* = 55.255, *p* = 0.028 < 0.05). At the same time, there was marginal significance between physical abuse and urgency (*F* = 33.397, *p* = 0.087), as well as between sexual abuse and sensation seeking (*F* = 33.191, *p* = 0.095 > 0.05).

**Table 5:** Multivariate ANOVA on impulsivity in crimes of passion offenders.

Factors	Pillai’s trace	*F*	*df*	SEx	*p*
Intercept	0.573	22.08	7	115	0.000***
BPD traits	0.034	0.578	7	115	0.773
Emotional abuse	0.019	0.324	7	115	0.942
Physical abuse	0.032	0.538	7	115	0.804
Sexual abuse	0.061	1.067	7	115	0.39
Neglect E	0.091	1.64	7	115	0.131
Neglect P	0.054	0.933	7	115	0.484
BPD × Emotional	0.049	0.844	7	115	0.553
BPD × Physical	0.065	1.143	7	115	0.342
BPD × Sexual	0.05	0.87	7	115	0.532
BPD × Neglect E	0.092	1.669	7	115	0.123
BPD × Neglect P	0.033	0.565	7	115	0.783

***p < 0.001.

**Table 6:** Effects of BPD traits and childhood maltreatment on impulsivity.

		Pillai’s trace	*F*	*df*	SEx	*p*
BPD traits	Attention	0.51	1	0.51	1.317	0.253
	Motor	0.039	1	0.039	0.101	0.752
	Nonplanning	1.595	1	1.595	2.642	0.107
	Premeditation	1.887	1	1.887	0.237	0.627
	Urgency	12.386	1	12.386	1.107	0.295
	Sensation seeking	0.882	1	0.882	0.074	0.787
	Perseverance	0.007	1	0.007	0.001	0.97
	UPPS total	33.032	1	33.032	0.569	0.452
Emotional abuse	Sensation seeking	19.376	1	19.376	1.617	0.206
Physical abuse	Premeditation	12.111	1	12.111	1.519	0.22
	Urgency	33.397	1	33.397	2.984	0.087
	UPPS total	141.973	1	141.973	2.445	0.121
Sexual abuse	Urgency	21.605	1	21.605	1.93	0.167
	Sensation seeking	33.919	1	33.919	2.83	0.095
	UPPS total	128.005	1	128.005	2.204	0.14
Neglect E	Attention	0.641	1	0.641	1.655	0.201
	Motor	0.589	1	0.589	1.53	0.219
	Nonplanning	0.827	1	0.827	1.37	0.244
	Urgency	55.255	1	55.255	4.937	0.028*
Neglect P	Motor	0.697	1	0.697	1.81	0.181

According to the Pillai’ trace test on impulsivity, dimensions with a value of *p* > 0.25 were hidden. *p < 0.05.

### Hierarchical regression analysis

A hierarchical regression analysis was employed to investigate the impacts of various factors on impulsivity among offenders of crimes of passion. The UPPS total score served as the dependent variable. Initially, demographic variables such as education level, age, occupation, and income level were evaluated. Subsequently, the primary effects of BPD score and childhood maltreatment score were assessed, followed by an examination of the interaction effect between the two. The results are provided in Table 7.

**Table 7:** Prediction of impulsivity in crimes of passion offenders.

	Adjusted *R*^2^	*F*	B	*β*	*t*	Sig
Model 1	0.043	3.023*				
Age			−0.097	−0.105	−1.753	0.081
Education			−0.758	−0.162	−2.267	0.024*
Profession			0.237	0.063	0.963	0.336
Income			−0.253	−0.044	−0.613	0.54
Model 2	0.225	12.883***				
BPD traits			1.41	0.423	7.167	0.000***
Childhood maltreatment			0.01	0.012	0.195	0.846
Model 3	0.226	11.085***				
BPD traits			0.974	0.292	1.442	0.151
Childhood maltreatment			−0.02	−0.025	−0.298	0.766
BPD × Childhood maltreatment			0.01	0.154	0.675	0.5

*p < 0.05, ***p < 0.001.

**Figure 2:**
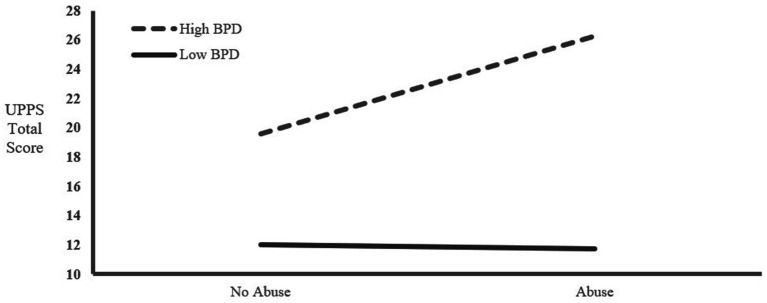
Interaction between high BPD trait and abuse on the UPPS total score.

In model 1, we had an *R*^2^ value of 0.043 (*F* = 3.023, *p* = 0.018 < 0.05), predicting a 4.3% variability in impulsivity. Among the various demographic variables, education significantly predicted impulsivity. We also found that inmates with higher educational levels had lower impulsivity (*t* = 2.267, *p* = 0.024 < 0.05), and impulsivity also decreased as age increased (*t* = 1.753, *p* = 0.081); both results were marginally significant. In the second model, we found an *R^2^* value of 0.225, which explained an additional 18.2% of the variability in impulsivity compared to the first model. Among the independent variables, the BPD score significantly predicted impulsivity (*t* = 7.167, *p* < 0.001), but childhood abuse did not predict impulsivity (*p* > 0.05). In the third model, we added the interaction between BPD and childhood maltreatment and found an *R^2^* value of 0.226, which predicted only an additional 0.1% increase in the volatility of impulsivity compared to the second model. However, in the third model, we found that neither childhood maltreatment nor the interaction between BPD and childhood maltreatment significantly predicted impulsivity (*p* > 0.05), and the BPD score that significantly predicted impulsivity in the second model also no longer predicted impulsivity (*p* > 0.05). To illustrate these relationships, the mean interaction graph is depicted in Figure 2.

## Discussion

This study aimed to examine the relationship between childhood maltreatment, BPD traits, and impulsivity in offenders of crimes of passion. The findings highlight a significant effect of childhood maltreatment and BPD traits on impulsivity in this population.

According to the chi-square test results, a significantly higher level of childhood maltreatment was observed in crimes of passion offenders compared to other offenders. Simultaneously, a higher BPD score was associated with a higher likelihood of childhood maltreatment, especially physical and emotional neglect. This aligns with past studies suggesting that childhood maltreatment is a significant factor leading to BPD (9, 11). Contrary to earlier research which mainly examined the impact of emotional and sexual abuse on BPD (23), the present study confirms recent meta-analyses indicating that childhood neglect (both physical and emotional) may also influence BPD symptoms in offender populations. This calls for further investigation and validation in different groups.

In the crimes of passion cohort, the BPD score was particularly strongly associated with childhood maltreatment, with the high BPD trait group scoring significantly higher on all types of childhood maltreatment except for sexual abuse than the low BPD trait group. Given previous research on the pathological mechanism of BPD (11, 15), it can be inferred that childhood maltreatment may increase the risk of BPD, potentially leading to a higher propensity to commit crimes of passion. Pearson correlation analysis further corroborated the significant association between childhood maltreatment, BPD traits, and crimes of passion, lending support to other studies’ findings (14).

The results from the independent sample t-test and correlation test on impulsivity in crimes of passion offenders align with our hypothesis. We anticipated that offenders with higher BPD traits would exhibit increased impulsivity, with this impulsivity positively correlated with childhood maltreatment. Notably, crimes of passion offenders displayed significantly higher scores on nonplanning and attention scales compared to other offenders. Additionally, those with high BPD traits scored significantly higher on urgency, premeditation, perseverance, and overall impulsivity scales than those with lower BPD traits. This aligns with previous studies linking BPD and impulsivity (14, 15) and suggests that higher BPD traits may contribute to increased impulsivity in crimes of passion offenders.

Our study extended the understanding of impulsivity in crimes of passion by considering a broader range of impulsivity dimensions, particularly those linked with crimes of passion and BPD. Although previous studies have mainly focused on urgency (16), our research indicated that attention and nonplanning could be central to impulsivity in crimes of passion.

In regard to the relationship between childhood maltreatment and impulsivity, our results only showed a moderate correlation. These findings are aligned with prior research, where a mild to moderate degree of correlation between childhood maltreatment and impulsivity was found (*r* values between 0.1 and 0.3) (10) (Shin et al., 2016) (24). This could explain why the validity of our study’s inference was affected by the statistical methods used. Moreover, significant differences were found in the associations between various types of abuse and different dimensions of impulsivity (10). As a result, our multivariate ANOVA indicated that the relationship between childhood maltreatment and impulsivity was predominantly driven by neglect.

Our multivariate ANOVA results also revealed a significant predictive effect of emotional neglect on urgency. Furthermore, high BPD traits and physical abuse were found to interact, affecting the UPPS total score. These results support the biosocial theory of BPD proposed in prior research (25, 26), and further substantiate the relationship between BPD, impulsivity, and crimes of passion (25, 26).

In our hierarchical regression analysis, we found that BPD traits could predict impulsivity in crimes of passion offenders, even without considering the interaction. However, when considering the interaction of BPD traits and childhood maltreatment, the prediction was no longer significant. This suggests that individuals with high BPD traits who commit crimes of passion are likely influenced by childhood maltreatment, leading to their impulsive criminal behavior. These results indicate that the interaction between childhood maltreatment and high BPD traits in crimes of passion offenders deserves further examination in future research. An enhanced understanding of how childhood maltreatment influences BPD symptoms, impulsivity, and behavioral control could be gained through pathophysiological mechanisms and neuropsychological measures of impulsivity, or via longitudinal experimental designs.

However, this study is not without its limitations. First, the cross-sectional design cannot determine the causal relationship between childhood maltreatment, BPD, and impulsivity. Potential confounding variables or reverse causality, such as neurobiological or environmental factors, may also impact offenders’ impulsivity. Second, due to the unique nature of prison samples, and the COVID-19 pandemic (2020–2021), prisons lacked professionals able to diagnose personality disorders. The limited number of participants and the difficulty in finding enough prisoners with clinically diagnosed BPD also affected our findings. As such, our study sample was not diagnosed with BPD but consisted of individuals with high BPD symptom scores on the MSI-BPD scale, which limited the inferences that could be drawn.

Another limitation was the potential underreporting or denial of abuse experiences due to cultural differences between China and the US, which could have affected the reported association between childhood maltreatment, BPD, and impulsivity. In China, high-pressure parenting and corporal punishment are common, and many children do not consider these practices abusive. This cultural context may have influenced participants’ responses.

Additionally, this study focused on BPD in crimes of passion offenders and did not explore other personality disorders, such as antisocial personality disorder. Although we hypothesized a stronger theoretical link between BPD, crimes of passion, childhood maltreatment, and impulsivity, other personality disorders may also play a significant role in criminal behavior. Future studies should investigate the influence of other personality disorders on crimes of passion and impulsivity, along with other mental disorders associated with childhood maltreatment and potentially affecting impulsivity, such as depression, anxiety, post-traumatic stress disorder, and substance abuse.

In conclusion, this study illustrated that childhood maltreatment and BPD traits significantly predict the impulsivity displayed by crimes of passion offenders. Moreover, an interaction was observed between physical abuse and high BPD traits, which contributed to an increase in the sense of urgency exhibited by these offenders. These findings contribute to the theoretical framework for developing interventions aimed at mitigating crimes of passion through the management of impulsivity.

The implications of our study are profound as it significantly broadens our understanding of the intricate relationships between childhood maltreatment, BPD traits, impulsivity, and crimes of passion. Despite the highlighted limitations, the insights gathered from this research underline the necessity for future studies to further probe into these associations, taking into account various types of personality disorders and other psychological conditions that may influence impulsivity. Such comprehensive exploration is imperative in refining interventions and preventive measures aimed at curbing crimes of passion, ultimately benefiting both individuals and society at large.

Furthermore, considering the culturally specific elements of childhood maltreatment and the subsequent impact on BPD traits and impulsivity, future research should strive to capture these nuances, aiding in the development of culturally sensitive intervention programs. As such, while this study provides a valuable stepping stone, it is clear that a more detailed understanding of these relationships is required. Through continued investigation, we can hope to provide a more inclusive and effective approach to addressing the pressing issue of crimes of passion, potentially leading to significant reductions in these violent offenses.

## Data availability statement

The original contributions presented in the study are included in the article/Supplementary material, further inquiries can be directed to the corresponding author.

## Ethics statement

The studies involving human participants were reviewed and approved by Academic Committee of the School of Psychological and Cognitive Sciences of Peking University Ethics Committee of the Shenzhen Prison, Guangdong, China. The patients/participants provided their written informed consent to participate in this study.

## Author contributions

MJ: study design, data collection, data analysis, and manuscript revision. ZW: data analysis and manuscript revision. YZ: data collection. JZ: manuscript revision. All authors contributed to the article and approved the submitted version.

## Funding

This work was supported by the grants from the School of Psychological and Cognitive Sciences of Peking University.

## Conflict of interest

The authors declare that the research was conducted in the absence of any commercial or financial relationships that could be construed as a potential conflict of interest.

## Publisher’s note

All claims expressed in this article are solely those of the authors and do not necessarily represent those of their affiliated organizations, or those of the publisher, the editors and the reviewers. Any product that may be evaluated in this article, or claim that may be made by its manufacturer, is not guaranteed or endorsed by the publisher.

## References

[1] Cimino L. The impulsive crime: aspects of psychopathology and psychodiagnosis In: Balloni A Sette R, editors. Handbook of research on trends and issues in crime prevention, rehabilitation, and victim support. Hershey, PA: IGI Global (2020). 79–99.

[2] Bornstein BH Wiener RL. Introduction to the special issue on emotion in legal judgment and decision making. Law Hum Behav. (2006) 30:115–8. doi: 10.1007/s10979-006-9023-2

[3] Guan M Li X Xiao W Miao D Liu X. Categorization and prediction of crimes of passion based on attitudes toward violence. Int J Offender Ther Comp Criminol. (2017) 61:1775–90. doi: 10.1177/0306624X16643501 27240818

[4] American Psychiatric Association. Diagnostic and statistical manual of mental disorders. 4th ed. tet rev. ed. Washington, DC: American Psychiatric Association (1994).

[5] Coccaro EF Lee R Mccloskey MS. Relationship between psychopathy, aggression, anger, impulsivity, and intermittent explosive disorder. Aggress Behav. (2014) 40:526–36. doi: 10.1002/ab.21536, PMID: 24760575

[6] Huang X. (2019). Development of crime of passion checklist (Unpublished Bachelor’s Thesis), Peking University.

[7] Moeller FG Barratt ES Dougherty DM Schmitz JM Swann AC. Psychiatric aspects of impulsivity. Am J Psychiatr. (2001) 158:1783–93. doi: 10.1176/appi.ajp.158.11.1783 11691682

[8] Butchart A Harvey A Mian M Furniss T. Preventing child maltreatment: A guide to taking action and generating evidence. Geneva: World Health Organization (2006).

[9] Greene CA Haisley L Wallace C Ford JD. Intergenerational effects of childhood maltreatment: a systematic review of the parenting practices of adult survivors of childhood abuse, neglect, and violence. Clin Psychol Rev. (2020) 80:101891. doi: 10.1016/j.cpr.2020.101891, PMID: 32745835 PMC7476782

[10] Liu RT. Childhood maltreatment and impulsivity: a meta-analysis and recommendations for future study. J Abnorm Child Psychol. (2019) 47:221–43. doi: 10.1007/s10802-018-0445-3, PMID: 29845580 PMC6269232

[11] Carr A Duff H Craddock F. A systematic review of the outcome of child abuse in long-term care. Trauma Violence Abuse. (2020) 21:660–77. doi: 10.1177/1524838018789154, PMID: 30033824

[12] Burnette ML Reppucci ND. Childhood abuse and aggression in girls: the contribution of borderline personality disorder. Dev Psychopathol. (2009) 21:309–17. doi: 10.1017/s0954579409000170, PMID: 19144235

[13] Paris J. The diagnosis of borderline personality disorder: problematic but better than the alternatives. Ann Clin Psychiatry. (2005) 17:41–6. doi: 10.1080/10401230590905461 15941030

[14] Krause-Utz A Erol E Brousianou AV Cackowski S Paret C Ende G. Self-reported impulsivity in women with borderline personality disorder: the role of childhood maltreatment severity and emotion regulation difficulties. Borderline Personal Disord Emot Dysregul. (2019) 6:6–14. doi: 10.1186/s40479-019-0101-8, PMID: 30873282 PMC6399941

[15] Scott LN Stepp SD Pilkonis PA. Prospective associations between features of borderline personality disorder, emotion dysregulation, and aggression. Personal Disord Theory Res Treat. (2014) 5:278–88. doi: 10.1037/per0000070, PMID: 24635753 PMC4099305

[16] Wang Z. (2020). Impulsivity among passionate criminals: the role of emotional interference (Unpublished master’s thesis), Peking University.

[17] Bernstein DP Fink L Handelsman L Foote J.eds. Childhood trauma questionnaire In: Assessment of family violence: a handbook for researchers and practitioners. Washington, DC: American Psychiatric Association (1998) doi: 10.1037/t02080-000

[18] Crow TM Levy KN. Adult attachment anxiety moderates the relation between self‐reported childhood maltreatment and borderline personality disorder features. Pers Ment Health. (2019) 13:239–249. doi: 10.1002/pmh.1468 31571424

[19] Whiteside SP Lynam DR Miller JD Reynolds SK. Validation of the UPPS impulsive behaviour scale: a four-factor model of impulsivity. Eur J Personal. (2005) 19:559–74. doi: 10.1002/per.556

[20] Coutlee CG Politzer CS Hoyle RH Huettel SA. An abbreviated impulsiveness scale constructed through confirmatory factor analysis of the Barratt impulsiveness scale version 11. Arch Sci Psychol. (2014) 2:1–12. doi: 10.1037/arc0000005 26258000 PMC4527550

[21] Lazuras L Ypsilanti A Powell P Overton P. The roles of impulsivity, self-regulation, and emotion regulation in the experience of self-disgust. Motiv Emot. (2019) 43:145–58. doi: 10.1007/s11031-018-9722-2

[22] Wang YY Leung F Zhong J. The adaptation of Mclean screening Instrument for borderline Personality disorder among Chinese college students. Chin J Clin Psych. (2008) 16:258–61. doi: 10.3969/j.issn.1005-3611.2008.03.010

[23] Salzman JP Salzman C Wolfson AN Albanese M Looper J Ostacher M. Association between borderline personality structure and history of childhood abuse in adult volunteers. Compr Psychiatry. (1993) 34:254–7. doi: 10.1016/0010-440x(93)90007-q, PMID: 8348804

[24] Shin SH Cook AK Morris NA McDougle R Groves LP. The different faces of impulsivity as links between childhood maltreatment and young adult crime. Prev Med. (2016) 88:210–217. doi: 10.1016/j.ypmed.2016.03.022 27083525 PMC5356379

[25] Paris J. The development of impulsivity and suicidality in borderline personality disorder. Dev Psychopathol. (2005) 17:1091–104. doi: 10.1017/s0954579405050510 16613432

[26] Paris J. Understanding self-mutilation in borderline personality disorder. Harv Rev Psychiatry. (2005) 13:179–85. doi: 10.1080/10673220591003614, PMID: 16020029

